# Efficacy and safety of dacomitinib in treatment-naïve patients with advanced NSCLC and brain metastasis: a multicenter cohort study

**DOI:** 10.1093/oncolo/oyaf079

**Published:** 2025-05-11

**Authors:** Puyuan Xing, Xingxiang Pu, Yu Zhou, Ziling Liu, Yu Yao, Jiayu Liu, Shouzheng Wang, Ying Hu, Junling Li, Lin Wu

**Affiliations:** Department of Medical Oncology, National Cancer Center/National Clinical Research Center for Cancer/Cancer Hospital, Chinese Academy of Medical Sciences & Peking Union Medical College, Beijing, 100021, People’s Republic of China; The Second Department of Thoracic Oncology, The Affiliated Cancer Hospital of Xiangya School of Medicine, Central South University/Hunan Cancer Hospital, Changsha, 410013,People’s Republic of China; The Second Department of Thoracic Oncology, The Affiliated Cancer Hospital of Xiangya School of Medicine, Central South University/Hunan Cancer Hospital, Changsha, 410013,People’s Republic of China; Department of Oncology, the First Hospital of Jilin University, Changchun, 130031, People’s Republic of China; Department of Oncology, the First Affiliated Hospital of Xi’an Jiaotong University, Xi’an, 710061, People’s Republic of China; Department of Medical Oncology, National Cancer Center/National Clinical Research Center for Cancer/Cancer Hospital, Chinese Academy of Medical Sciences & Peking Union Medical College, Beijing, 100021, People’s Republic of China; The Department of Oncology, Beijing Chest Hospital, Beijing, 101149, People’s Republic of China; The Department of Oncology, Beijing Chest Hospital, Beijing, 101149, People’s Republic of China; Department of Medical Oncology, National Cancer Center/National Clinical Research Center for Cancer/Cancer Hospital, Chinese Academy of Medical Sciences & Peking Union Medical College, Beijing, 100021, People’s Republic of China; The Second Department of Thoracic Oncology, The Affiliated Cancer Hospital of Xiangya School of Medicine, Central South University/Hunan Cancer Hospital, Changsha, 410013,People’s Republic of China

**Keywords:** non-small cell lung cancer, EGFR, brain metastasis, dacomitinib, treatment

## Abstract

**Background:**

The data for dacomitinib, a second-generation EGFR-TKI, treating patients with advanced non-small cell lung cancer (NSCLC) and brain metastasis was lacking. This study aimed to explore the efficacy and safety of dacomitinib in treating EGFR-mutated advanced NSCLC with brain metastasis in first-line settings.

**Methods:**

Eligible patients were treatment-naïve advanced NSCLC patients with ≥1 brain metastasis no less than 5 mm treated with dacomitinib. The primary endpoint was intracranial objective response rate (ORR). Secondary endpoints included intracranial and extracranial progression-free survival (PFS), overall survival (OS), intracranial and extracranial ORR, disease control rate (DCR), and safety. The response was evaluated per modified Response Evaluation Criteria in Solid Tumors (mRECIST) and RANO-BM (Response Assessment in Neuro-Oncology Brain Metastases) criteria.

**Result:**

Between July 2nd, 2019, and September 30th, 2022, a total of 87 treatment-naïve patients with advanced NSCLC and brain metastasis treated with dacomitinib from four hospitals were included. The data cutoff date was March 24th, 2023, and the median duration of follow-up time was 17.5 months (range 1.6-34.7 months). Based on mRECIST criteria, for all the 87 patients with evaluable brain metastasis, the iORR was 89.7% (95%CI, 81.3%-95.2%) and iDCR was 97.7% (95%CI, 91.9-99.7%), with 42 patients achieving CR, 36 patients achieving PR, and 7 patients maintaining SD. Based on RANO-BM criteria, the iORR was 71.3% (62/87, 95%CI 60.6%-80.5%) and iDCR was 97.7% (85/87, 95%CI, 91.9%-99.7%), with 42 patients achieving CR, 20 patients achieving PR, and 23 patients maintaining SD (Table). Median iPFS was 26.0 (95%CI, 20.7-31.4) months, and the 1-year and 2-year iPFS rate were 68.9% and 51.5%, respectively. Of 75 patients with evaluable extracranial lesions, 2 patients achieved CR (2.7%), the systemic ORR was 73.8% (95%CI 63.1%-82.8%) and DCR was 96.4% (89.9%-99.3%) (Table). Systemic median PFS was 14.0 (95%CI 11.1-16.9) months and median OS was 34.0 (95%CI 28.0-39.9) months. Overall, 86 of 87 (98.9%) patients experienced adverse events (AEs) of any grade. The most common (≥20%) AEs including rash (89.7%), oral ulcer (74.2%), diarrhea (67.8%), and paronychia (59.8%). Most of the AEs were grade 1 or grade 2 and no patients died due to severe AEs.

**Conclusions:**

Dacomitinib showed promising efficacy and a manageable safety profile for advanced NSCLC with brain metastasis harboring EGFR mutation in the first-line treatment.

**Implications for practice**

- Baseline brain metastasis occurred in about 10% of the non-small cell lung cancer (NSCLC) patients and cause rather poor prognosis.
- In this study, we explored the efficacy and safety for dacomitinib treating treatment-naïve advanced NSCLC patients harboring EGFR mutation with brain metastasis.
- The intracranial objective response rate was 89.7% and intracranial disease control rate was 97.7%.
- Median intracranial progression-free survival (PFS) was 26.0 months. Systemic median PFS was 14.0 months and median overall survival was 34.0 months.
- Overall, dacomitinib treatment was tolerable with most adverse events being grade 1 to 2.
- Dacomitinib showed promising efficacy and a manageable safety profile for advanced NSCLC with brain metastasis harboring EGFR mutation in the first-line treatment and could be an essential treatment option for these patients.

## Introduction

Lung cancer is one of the most common cancers, of which non-small cell lung cancer (NSCLC) accounts for approximately 85%.^1^ For patients with advanced NSCLC harboring epidermal growth factor receptor (*EGFR*) mutations, *EGFR* tyrosine kinase inhibitor (EGFR-TKI) was the standard treatment and provided many treatment options. Baseline brain metastasis occurred in about 10% of the NSCLC patients,^2,3^ and about 20%-30% of patients with NSCLC developed brain metastasis during treatment.^4-6^ The efficacy of different EGFR-TKIs in treating patients with brain metastasis should be further explored as the prognosis of patients with brain metastasis was rather poor.

Dacomitinib, a second-generation EGFR-TKI, has been approved for the treatment of advanced NSCLC harboring *EGFR* exon 19 (19 del) and Leu858Arg point mutation (L858R) in exon 21 mutation. In ARCHER 1050 study, the randomized, open-label, phase III trial, dacomitinib showed a significant improvement in progression-free survival (PFS) and overall survival (OS) compared to gefitinib.^7,8^ However, in this study, patients with baseline brain metastasis were excluded, which led to limited data on the efficacy and safety of dacomitinib in advanced NSCLC with brain metastasis.

Preclinical and clinical studies showed that dacomitinib had potential efficacy for patients with brain tumors including glioblastoma which indicated the capability of dacomitinib to penetrate the blood-brain barrier.^9-11^ The first case of one patient with multiple brain metastatic lesions from *EGFR*-mutated NSCLC treated with dacomitinib as the sixth-line therapy was reported by Kudo et al and all of the brain lesions were undetectable after 2-months of treatment.^12^ The potential efficacy of dacomitinib in brain metastasis in NSCLC patients in the first-line settings was further explored in 2 cohort studies, with the intracranial objective response rate (ORR) ranging from 87.5% to 92.9% and intracranial DCR 100%.^13,14^ However, these 2 studies were with relatively small samples (8 and 14 central nervous system [CNS] response evaluable patients in these 2 studies, respectively). Also, CNS response was evaluated by different criteria in these 2 studies, and a larger-scale cohort study with consistent response evaluation criteria is warranted to further explore the efficacy and safety of dacomitinib in treating NSCLC with brain metastasis.

In this study, we conducted a multicenter ambispective cohort study aiming to provide more evidence for the application of dacomitinib treating advanced NSCLC harboring EGFR mutation in a real-world setting.

## Materials and method

### Study design and eligibility criteria

This study is an ambispective cohort study aiming to evaluate the efficacy and safety of dacomitinib in treating treatment-naïve advanced NSCLC with harboring *EGFR* mutation with brain metastasis. Eligible patients were pathologically confirmed stage Ⅳ NSCLC with *EGFR* mutation (exon 19 deletion or Leu858Arg and other uncommon *EGFR* mutations); with at least one measurable intracranial target lesion with the long axis greater or equal to 5 mm; Eastern Cooperative Oncology Group (ECOG) Performance Status (PS) 0-2; age over 18 years old and with adequate organ and bone marrow function; not receiving prior brain metastasis surgery or radiotherapy; receiving dacomitinib as first-line therapy. Exclusion criteria included: concomitant cancer or serious disease; previous exposure to any other EGFR-TKIs, and radiotherapy or chemotherapy.

*EGFR* mutation detection was performed in the core laboratory of each institution and analyzed using the next‐generation sequencing (NGS) method. Accompanying mutations (defined as mutations detected other than *EGFR*) and *EGFR* compound mutations (defined as the presence of more than one *EGFR* mutation, either common or uncommon) were also recorded.

This study protocol was confirmed by the Ethics Committee of all enrolled centers and conducted by the Declaration of Helsinki. Each patient participating in the study signed an informed consent form.

### Treatment plan

All patients were treated with dacomitinib. Initial doses of dacomitinib were 30 mg (for elderly patients or patients with inferior ECOG PS status assessed by clinicians) or 45 mg per day, administered orally once a day until disease progression or intolerant side effects developed. When grade 3-4 adverse events (AEs) occurred, treatment would be suspended until patients recovered to no more than grade 1 AE and the dose of dacomitinib should be adjusted to the lower level (45 mg adjusted to 30 mg and 30 mg adjusted to 15 mg) afterward. For patients with grade 2 AEs, dose adjustment was not necessary unless grade 2 AEs recurred. Brain radiotherapy at baseline was permitted only if patients were with symptomatic brain metastasis.

### Response assessment and evaluation of adverse reactions

Computed tomography (CT) scans or magnetic resonance imaging (MRI) were applied to evaluate treatment response before and during dacomitinib treatment. CT scan covering the neck, chest, abdomen, and pelvis was required for systemic assessment. MRI of the brain was required at baseline and each tumor assessment. Target lesions were assessed every 6-8 weeks. The intracranial response was evaluated per modified form of Response Evaluation Criteria in Solid Tumors (mRECIST)^^15,16^^ and RANO-BM (Response Assessment in Neuro-Oncology Brain Metastases) criteria.^17^ In the modified RECIST, up to 5 intracranial and up to 5 extracranial target lesions were included; intracranial target lesions of no less than 5 mm in diameter were allowed. RANO-BM allows the use of a 5 mm cutoff if the MRI slice thickness is equal to or less than 1.5 mm. In this study, the slice thicknesses of the MRI were 1.5 mm, and the 5 mm cutoff was adopted. In RANO-BM criteria, complete response (CR) was defined as no evidence of disease, partial response (PR) was defined as a decrease by >50% in the summed product of orthogonal diameters, and progressive disease (PD) was defined as an increase by >25% in the summed product of orthogonal diameters. The remaining situation was defined as stable disease (SD). Systemic response was evaluated according to Response Evaluation Criteria in Solid Tumors (RECIST) version 1.1.^15^ Telephone follow-up was conducted every 3 months. The last follow-up date for this study was February 22nd, 2023.

Adverse events were recorded and assessed using Common Terminology Criteria for Adverse Events (CTCAE) version 4.03.

### Outcomes

The primary endpoint was intracranial objective response rate (iORR, defined as the percentage of patients who achieved CR and PR for intracranial lesions). Secondary endpoints included intracranial progression-free survival (iPFS, defined as the time interval from treatment to intracranial lesion progression or death), systemic progression-free survival (PFS, defined as the time from the date of treatment to that of death or disease progression based on RECIST criteria), extracranial objective response rate (ORR, defined as the percentage of patients who achieve CR and PR for systemic lesions), intracranial and extracranial disease control rate (DCR, defined as the percentage of patients who achieved CR, PR, and SD), overall survival (OS, defined as the time interval from treatment to death of any cause) and safety. Of note, extracranial disease progression was managed as censored for the analysis of intracranial PFS. When patients experienced brain oligo-progression and took the brain radiotherapy while still using dacomitinib, these patients were considered as disease progression and defined as events for the analysis of intracranial PFS.

### Statistical analysis

SPSS software (version 26, IBM) was used for all the statistical analyses. GraphPad Prism 9 software (GraphPad Software) was used for visualization. Survival data were analyzed using the Kaplan-Meier method. *P*-value < .05 indicates a statistically significant difference.

## Results

### Baseline characteristics

**Figure 1.**
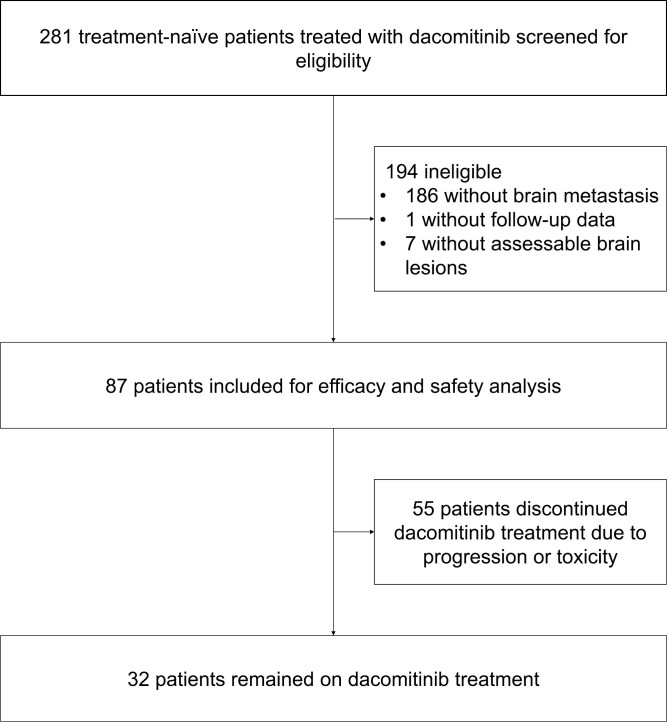
Diagram of the study enrollment and treatment procedure.

Between July 2nd, 2019, and September 30th, 2022, of 281 treatment-naïve patients with advanced NSCLC treated with dacomitinib screened for eligibility, a total of 87 patients from 4 hospitals were enrolled and included in the efficacy and safety analysis. The data cutoff date was March 24th, 2023, and the median duration of follow‐up time was 17.5 months (range 1.6-34.7 months). On the data cutoff date, 32 patients remained on the treatment of dacomitinib. The diagram is shown in Figure 1. The baseline characteristics of the 87 patients are shown in Table 1. Enrolled patients were between 41 and 84 years old and most (55/87, 63.2%) of them were female. For the *EGFR* mutation type, 20 of 87 (23.0%) patients were harboring *EGFR* 19 del mutation, 58 of 87 (66.7%) patients harboring L858R mutation, and the rest 9 of 87 (10.3%) were with uncommon *EGFR* mutations, of whom 5 patients with *EGFR* L861Q mutation, 2 with G719X mutation, one with E709K mutation, and one with L859R mutation. A total of 3 (3.4%) patients were harboring *EGFR* compound mutation and 51 (58.6%) patients had accompanying mutations.

**Table 1.** Characteristics of enrolled patients and treatment (*N* = 87).

Characteristics	*n* (%)
Median age, years (range)	58 (41-84)
Gender	
Male	32 (36.8)
Female	55 (63.2)
ECOG PS score	
0	30 (34.5)
1	48 (55.2)
2	9 (10.3)
Smoking status	
Nonsmoker	61 (70.1)
Smoker/former smoker	26 (29.9)
Histology	
Adenocarcinoma	83 (95.4)
Adenosquamous carcinoma	2 (2.3)
Others	2 (2.3)
Metastatic sites	
Single site metastasis	4 (4.6)
Multiple site metastasis	83 (95.4)
*EGFR* mutation type	
19 del mutation	20 (23.0)
21 L858R mutation	58 (66.7)
others	9 (10.3)
PD-L1 expression	
< 1%	31 (35.6)
1 - 49%	11 (12.6)
≥ 50%	5 (5.7)
unknown	40 (46.0)
unknown	40 (46.0)
*EGFR* compound mutation	
Yes	3 (3.4)
No	84 (96.6)
Accompanying mutation	
yes	51 (58.6)
no	36 (41.4)
Initial dosage of dacomitinib	
15 mg	1 (1.5)
30 mg	55 (64.3)
45 mg	31 (35.6)

*Note*: Data are presented as number (%) unless otherwise indicated.

Abbreviations: ECOG PS, Eastern Cooperative Oncology Group performance status; *EGFR*, epidermal growth factor receptor; PD-L1, programmed cell death-ligand 1.

### Treatment procedure

For the initial dose, 64.3% (56 of 87) of the patients started with 30 mg per day dacomitinib while the rest 35.6% (31 of 87) started with 45 mg per day dacomitinib. Overall, 26 (29.9%) patients experienced the dose reduction. Five (5.7%) patients experienced brain radiotherapy at baseline for symptomatic brain metastasis.

### Efficacy

#### Efficacy analysis of brain metastasis

**Figure 2.**
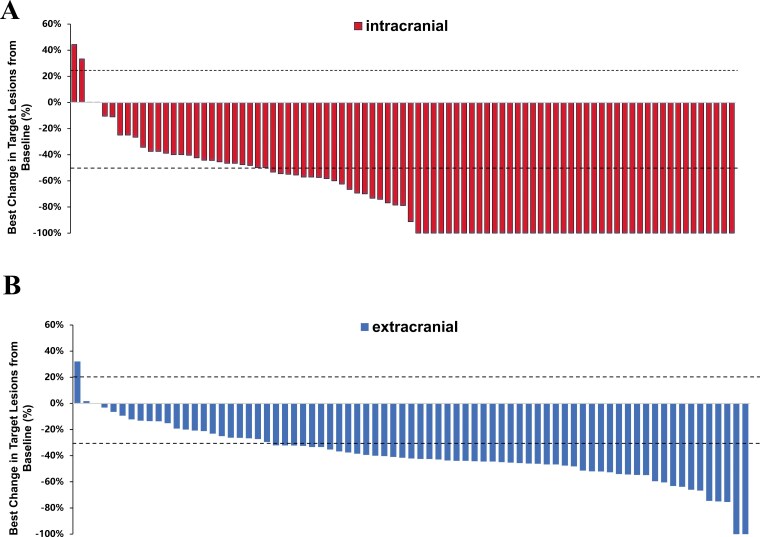
Best percentage change in target lesion size from baseline. (A) intracranial lesions; (B) systemic lesions. *Note*: The upper dashed line at +20% represents the threshold for progressive disease and the lower dashed line at −30% represents the boundary for partial response per mRECIST 1.1 criteria.

Based on mRECIST criteria, for all the 87 patients with evaluable brain metastasis, the iORR was 89.7% (95%CI, 81.3%-95.2%) and iDCR was 97.7% (95%CI, 91.9%-99.7%), with 42 patients achieving CR, 36 patients achieving PR and 7 patients maintaining SD. Based on RANO-BM criteria, the iORR was 71.3% (62/87, 95%CI 60.6%-80.5%) and iDCR was 97.7% (85/87, 95%CI ,91.9%-99.7%), with 42 patients achieving CR, 20 patients achieving PR and 23 patients maintaining SD (Table 2). The waterfall plot showing the remission of all evaluable intracranial lesions was demonstrated in Figure 2A. For 20 patients harboring 19 del mutation the intracranial ORR was 75.0% (95%CI, 50.9%-91.3%), DCR was 100% (95%CI, 83.2%-100%) while for the 58 patients harboring L858R mutation, the intracranial ORR was 70.7% (95%CI, 57.3%-81.9%), DCR was 96.6% (95%CI, 88.1%-99.6%). Especially, a total of 9 patients with uncommon *EGFR* mutation were included. Four patients achieved intracranial CR, 2 patients achieved intracranial PR and the remaining 3 patients stayed SD. The intracranial ORR was 66.7% (95%CI, 29.9%-92.5%) and the intracranial DCR was 100% (95%CI, 66.4%-100%).

**Table 2.** Intracranial and systemic efficacy.

	Intracranial efficacy (*N* = 87)		Systematic efficacy (*N* = 75)
	per mRECIST criteria	per RANO-BM criteria	
Complete response, n (%)	42 (48.3)	42 (48.3)	2 (2.7)
Partial response, n (%)	36 (41.8)	20 (23.0)	51 (68.0)
Stable disease, n (%)	7 (8.0)	23 (26.4)	21 (28.0)
ORR (95%CI)	89.7 (81.3-95.2)	71.3 (60.6-80.5)	73.8 (63.1-82.8)
DCR (95%CI)	97.7 (91.9-99.7)	97.7 (91.9-99.7)	96.4 (89.9-99.3)
PFS, median (95%CI)	26.0 (22.5-29.5)		14.0 (11.1-16.9)
One-year PFS rate	68.9%		53.1%
Two-year PFS rate	51.5%		24.1%

Abbreviations: ORR, objective response rate; DCR, disease control rate; PFS, progression-free survival.

**Figure 3.**
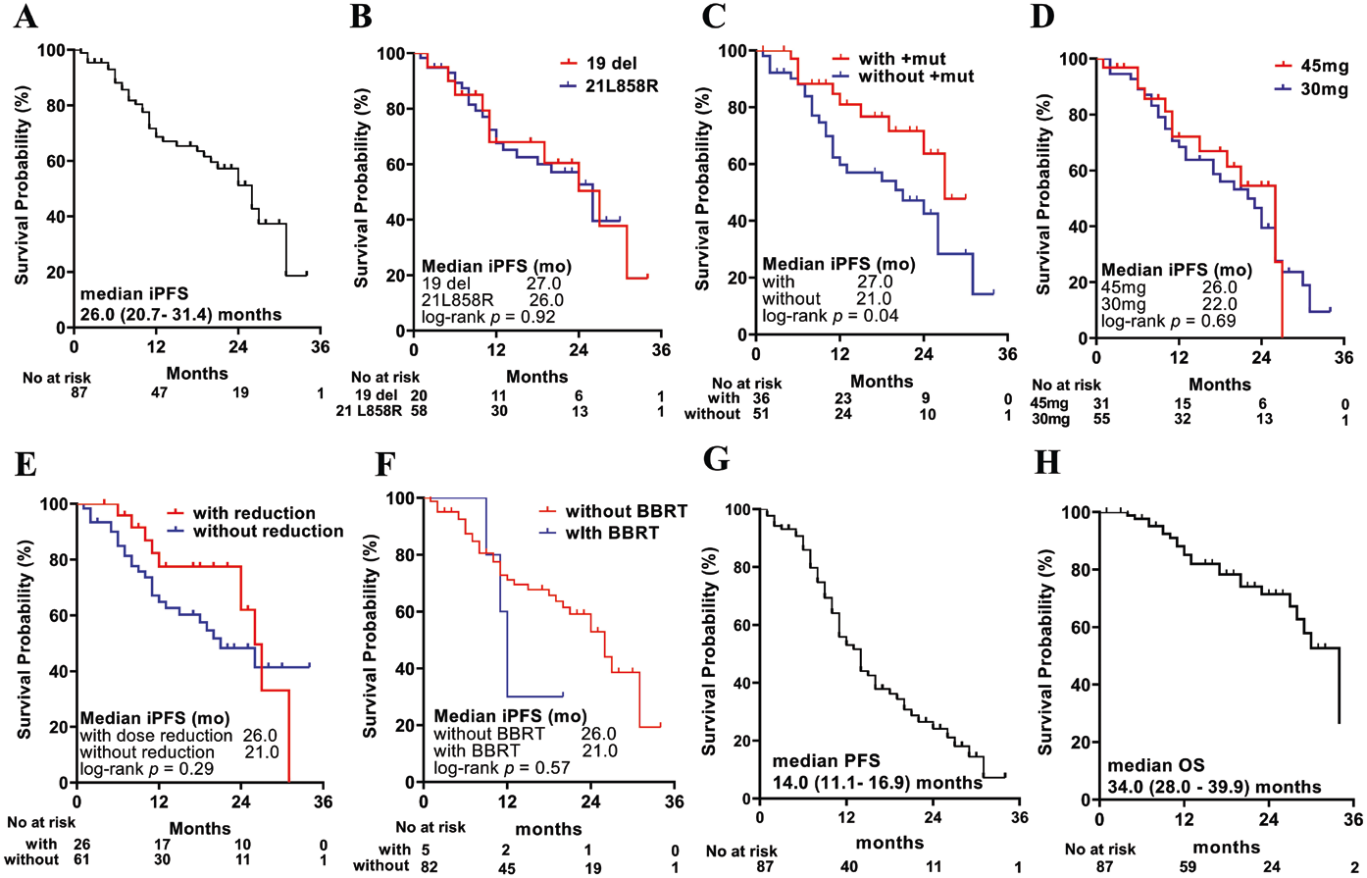
Kaplan-Meier plots for (A)overall intracranial progression-free survival (iPFS); (B)iPFS of major mutation types; (C) iPFS of with or without accompanying mutation; (D)iPFS of different initial dose groups; (E) iPFS of patients with or without dose reduction; (F) iPFS of patients with or without baseline brain radiotherapy; (G) systemic progression-free survival; (H) systemic overall survival. Abbreviations: +mut, accompanying mutation; BBRT, baseline brain radiotherapy.

Overall, median iPFS was 26.0 (95%CI, 20.7-31.4) months, and the 1-year and 2-year iPFS rates were 68.9% and 51.5%, respectively (Figure 3A). For patients harboring 19 del mutation, the median iPFS was 27.0 (95%CI 16.7 – 37.3) months, while the iPFS was 26.0 (95%CI, 19.2-32.8) months for patients harboring L858R mutation (*P* = .92, Figure 3B). Compared to patients without accompanying mutations, those with accompanying mutations showed better intracranial lesion-related PFS (*P* = .04) (Figure 3C) and there was no significant difference found for different initial dose groups (*P* = .69) (Figure 3D). For patients without dose reduction, the median iPFS was 21.0 (95%CI, 13.5–28.5) months, while the median iPFS for patients with dose reduction was 26.0 (95%CI, 23.0-28.9) months (*P* = .29) (Figure 3E). There was no significant difference in median intracranial lesion-related PFS for patients who received baseline brain radiotherapy compared to those not, with the median iPFS was 26.0 (95%CI, 20.7-31.3) months and 12.0 (95%CI, 10.4-13.6) months (*P* = .57), respectively (Figure 3F).

#### Systemic efficacy analysis

Based on mRECIST 1.1 criteria, of 75 patients with evaluable extracranial lesions, 2 patients achieved CR (2.7%), 51 patients achieved PR (68.0%), and stable disease was seen in 21 (28.0%) patients. The systemic ORR was 73.8% (95%CI, 63.1%-82.8%) and DCR was 96.4% (89.9%-99.3%) (Table 2). The waterfall plot of systemic remission was shown in Figure 2B. At data cut-off date, 58 patients were assessed as disease progression and 23 patients died. Median PFS was 14.0 (95%CI 11.1-16.9) months and median OS was 34.0 (95%CI 28.0-39.9) months. One-year PFS and OS rate were 53.1% and 85.1%, respectively. Two-year PFS rate and OS rate were 24.1% and 71.5%, respectively (Figure 3G and 3H).

### Safety

Overall, 86 of 87 (98.9%) patients experienced adverse events (AEs) of any grade. The most common (≥20%) AEs included rash (89.7%), oral ulcer (74.2%), diarrhea (67.8%), and paronychia (59.8%). Most of the AEs were grade 1 or grade 2 and no patients died due to severe AEs. Grade 3 or higher AEs included rash (11.4%), paronychia (4.6%), oral ulcer (3.4%), and diarrhea (3.4%). A total of 26 patients experienced dose reduction including 15 patients who started with 45 mg per day and 11 with 30 mg per day, and one patient discontinued dacomitinib treatment due to AEs. The safety profile is summarized in Table 3.

**Table 3.** Treatment-emergent adverse events (*N* = 87). Abbreviations: AST, aspartate transaminase; ALT, alanine aminotransferase.

Adverse events	Any grade (*n*, %)	Grade 1 (*n*, %)	Grade 2 (*n*, %)	≥Grade 3 (*n*, %)
Rash	78 (89.7)	35 (40.2)	33 (37.9)	10 (11.4)
Oral ulcer	63 (74.2)	37 (42.5)	23 (26.4)	3 (3.4)
Diarrhea	59 (67.8)	40 (46.0)	16 (18.4)	3 (3.4)
Paronychia	52 (59.8)	33 (37.9)	15 (17.2)	4 (4.6)
Interstitial pneumonia	5 (5.7)	2 (2.3)	3 (3.4)	0
Musculoskeletal pain	3 (3.4)	2 (2.3)	1 (1.1)	0
AST or ALT elevated	3 (3.4)	3 (3.4)	0	0
Chilly	2 (2.3)	2 (2.3)	0	0
Nausea	2 (2.3)	2 (2.3)	0	0
Asthenia	2 (2.3)	2 (2.3)	0	0
Alopecia	2 (2.3)	2 (2.3)	0	0

### Subsequent treatment

At the data cutoff date, 58 of 87 patients experienced systemic disease progression, 37 with local progression, and 21 with distant progression. A total of 36 patients experienced intracranial disease progression. Of the 58 patients with disease progression, 26 patients took third-generation EGFR-TKI as subsequent treatment. 13 patients received immunotherapy combined with platinum-based chemotherapy with or without bevacizumab. Five patients took brain radiotherapy and continued dacomitinib treatment. Two patients died without subsequent treatment and the remaining 12 patients refused the above treatment and took Chinese traditional herbs as subsequent treatment.

## Discussion

In this multi-center ambispective cohort study, dacomitinib showed promising activity and a manageable safety profile in advanced NSCLC with brain metastasis, with an iORR of 71.3% and iDCR of 97.7%. Median intracranial lesion-related PFS was 26.0 months and the 1-year PFS rate was 68.9%. Systemic ORR was 73.8%, DCR was 96.4% and median PFS and OS were 14.0 months and 34.0 months, respectively. The efficacy results of our study were comparable to the result of the ARCHER 1050 study and former 2 retrospective cohort studies,^7,8,13,14^ which indicated the reliability of the efficacy of dacomitinib in treating NSCLC with brain metastasis. To the best of our knowledge, our study is the largest multi-center cohort study reporting the efficacy and safety data of dacomitinib in treating advanced NSCLC with brain metastasis.

Baseline brain metastasis happened in about 10% of treatment-naïve advanced NSCLC. For patients harboring *EGFR* mutation, the efficacy of different EGFR-TKIs varied and usually these patients had poor prognosis compared to those without brain metastasis.^18^ Selecting the optimal treatment for these patients should be considered. For the first-generation of EGFR-TKI, gefitinib alone demonstrated an intracranial response rate of 87.8% (according to RECIST) for patients with *EGFR*-mutant advanced NSCLC and brain metastasis in the first-line setting.^19^ With more and more preclinical studies showing that second- and third-generation EGFR-TKIs had the more potent ability of CNS concentration and penetration rate, the activity of second- and third-generation EGFR-TKIs was further explored in the prospective or retrospective studies. In LUX-Lung 7 study, a total of 51 patients with baseline brain metastasis were included. The median systemic PFS was 7.2 months for the afatinib group while the gefitinib group showed a median PFS of 7.4 months (HR = 0.76 [0.55-1.06], *P* = 0.93).^20^ Due to the small number of patients and lack of efficacy information specific to brain metastasis, this subgroup analysis result should be interpreted with caution. A study by Jung et al was conducted in a real-world setting with a larger sample size. In this study, a total of 198 treatment-naïve patients with *EGFR*-mutant advanced NSCLC and initial brain metastasis treated with gefitinib, erlotinib, and afatinib were included and the CNS response rate of gefitinib, erlotinib, and afatinib were reported as 64.7%, 68.2%, and 72.9%, respectively (*P* = 0.78). For CNS-PFS, afatinib showed significantly prolonged PFS, with 17.3 months for gefitinib, 12.4 months for erlotinib, and 23.3 months for afatinib (*P* < 0.001).^21^ Other retrospective studies analyzed brain metastatic lung adenocarcinoma patients treated with afatinib monotherapy. The overall intracranial ORR for afatinib monotherapy group was consistent, ranging from 81.1% to 81.8%.^22,23^

Osimertinib has also showed promising efficacy in the first-line setting. Preclinical studies indicated that osimertinib could achieve significant penetration in the brain than other EGFR-TKIs.^24,25^ In the FLAURA study, subgroup analysis showed that for patients with CNS metastasis at trial entry, osimertinib showed favorable PFS, OS, and CNS-PFS. CNS objective response rates for osimertinib were 91% (per RECIST criteria) in patients with no less than one measurable CNS lesion and the median CNS PFS was not reached with a median follow-up of 15.0 months.^26^ In another cohort study, the efficacy of 102 treatment-naïve patients with brain metastasis treated with osimertinib and 91 patients treated with first-generation EGFR-TKI were evaluated.^27^ Intracranial ORR was 68% with osimertinib and 50% with first-generation EGFR-TKIs. Significantly prolonged systemic OS, as well as intracranial PFS, were found in the osimertinib group compared with first-generation EGFR-TKIs. In a retrospective study by Kuo et al, patients with brain metastasis treated with gefitinib, erlotinib, afatinib, or osimertinib had significantly different systemic PFS (median PFS: 7.5, 10.0, 14.8 months or not reached, respectively, *P* = .0081), while the initial intracranial treatment response was similar (75%, 75%, 80%, or 86%, respectively, *P* = .875).^28^ In our study, the median systemic PFS was 14.0 months. Meanwhile, the intracranial ORR and DCR in our study were 89.7% and 97.7% per mRECIST criteria and 71.3% and 97.7% according to RANO-BM criteria, respectively. Median intracranial PFS was 26.0 months, which were comparable with the previous data of afatinib and osimertinib. The results of our study demonstrated the potential of dacomitinib in treating advanced NSCLC patients with brain metastasis. Also, for patients with uncommon *EGFR* mutation, the intracranial response in our study was comparable with those harboring sensitive *EGFR* mutations. Our study included patients with uncommon *EGFR* mutations were predominantly harboring L861Q and G719X mutations, which were considered as P-loop and αC-helix compressing (PACC) mutations suggested by Robichaux et al. in a previous study.^29^ Preclinical study showed that PACC mutations are robustly sensitive to second-generation TKIs and our study further provided data for the application of second-generation TKI dacomitinib treating patients harboring PACC mutations with brain metastasis, though a larger scale of prospective cohort study still warranted for validation.

Except for osimertinib, some ongoing trials have investigated the efficacy of other third-generation EGFR-TKIs in treating patients with EGFR mutation as well as brain metastasis. In the phase Ⅲ randomized AENEAS trial, 115 patients with CNS metastasis were enrolled and in the subgroup analysis. Aumolertinib showed favorable systemic PFS compared to gefitinib (median systemic PFS 15.3 months for aumolertinib and 8.2 months for gefitinib, HR = 0.38 [0.24-0.60]).^30^ Similarly, in the phase III FURLONG study, a total of 121 patients with CNS metastasis were included. In patients with CNS metastasis, the median systemic PFS was 18.0 months for the furmonertinib group and 12.4 months for the gefitinib group (HR = 0.5 [0.32-0.80], *P* = .0028).^31^ Although subgroup analysis of these 2 large-scale prospective clinical studies showed the systemic survival benefit from aumolertinib and furmonertinib, CNS-specific data was lacking and warranted to further validate the efficacy of these 2 third-generation EGFR-TKIs for brain metastasis.

Regarding safety, commonly reported AEs of second- and third-generation EGFR-TKIs included rash, dry skin, diarrhea, paronychia, and interstitial lung disease. Previous meta-analysis pooled safety data from randomized controlled trials and results indicated that second-generation EGFR-TKI afatinib and dacomitinib might have a greater probability of grade 3 or higher AEs and third-generation EGFR-TKI osimertinib has mild toxicity profile.^32,33^ For specific AEs, among the different EGFR-TKIs, dacomitinib was associated with the highest risk of paronychia and dry skin and diarrhea was also another AE that needed to be noted. Osimertinib could have more events in cardiac function. In our study, dacomitinib showed a tolerable safety spectrum with most of the AEs grade 1 or grade 2. Only one patient discontinued dacomitinib treatment due to AEs. The manageable toxicity of dacomitinib in the real-world could be associated with the dose-titration strategy in clinical practice. In our study, the initial dose of dacomitinib was predominately 30 mg per day, although the recommended initial dose was 45 mg per day. According to previous reports, when taking the recommended initial dose of dacomitinib 45 mg per day, the treatment-related adverse events were frequently observed and about 66% of patients had experienced dose reduction.^34^ Also, the study showed that median PFS and OS were similar in all dacomitinib-treated patients and dose-reducing patients.^34^ Therefore, the dose-titration strategy was explored in the clinical trial and applied in clinical practice to better manage the toxicity of dacomitinib without hampering the efficacy, with an initial dose at 30 mg per day with escalation to 45mg per day if grade ≤ 1 gastrointestinal toxicity appeared (NCT04027647). In our study, we compared the intracranial efficacy between patients with dose reduction and those without, as well as different initial dose groups. The intracranial PFS of these 2 different groups were close without significant differences, which indicates the different initial dose and dose reduction of dacomitinib did not diminish the intracranial therapeutic effect.

There were some limitations of our study. This is an ambispective cohort study that collected data both retrospectively and prospectively, which might cause selection bias inevitably. A prospective, single-arm, phase Ⅱ study of dacomitinib for EGFR-mutated NSCLC with brain metastasis is ongoing (NCT04339829). Also, this was a single-arm cohort study. Further head-to-head trials are warranted.

## Conclusion

Dacomitinib showed promising efficacy and a manageable safety profile for advanced NSCLC with brain metastasis harboring EGFR mutation in the first-line treatment. The results of this study indicated that dacomitinib could be an essential treatment option for these patients.

## Data Availability

The original data can be acquired from the corresponding authors under reasonable requirement.
